# Examination of gene expression in *Mu* insertion alleles of *oil yellow1* during vegetative development in *Zea mays*

**DOI:** 10.17912/micropub.biology.001643

**Published:** 2025-08-05

**Authors:** Sophia Mihalatos, Kevin Tompkins, Victoria Busch, Olivia Fanuele, Nuranjalie Outar, Aaron N. Saran, Pukhraj Kaur, Daniel Koulta, Gurninder Ahluwalia, Vanessa Froehlich, Stephanie Lee, Sicong Wang, Dafang F. Wang

**Affiliations:** 1 Biology, Hofstra University, Hempstead, New York, United States

## Abstract

The *oil yellow1* ( *oy1* ) gene encodes the I subunit of magnesium chelatase, a key enzyme in chlorophyll biosynthesis in Z *ea mays* . Using Uniform *Mu* insertion lines, we examined how *Mu* insertions influence OY1 expression across vegetative development. Transcription remained unchanged during juvenile stages (V3-V5) but was consistently upregulated in adult stages (V7-V9). Related genes CHLD1 and CHLH1 showed similar patterns. Although transposon insertions often disrupt gene expression, our findings show they can also enhance transcription in a stage-specific manner.

**Figure 1. Gene Expression and Phenotype of Mu Insertions in oy1 in Zea mays:**
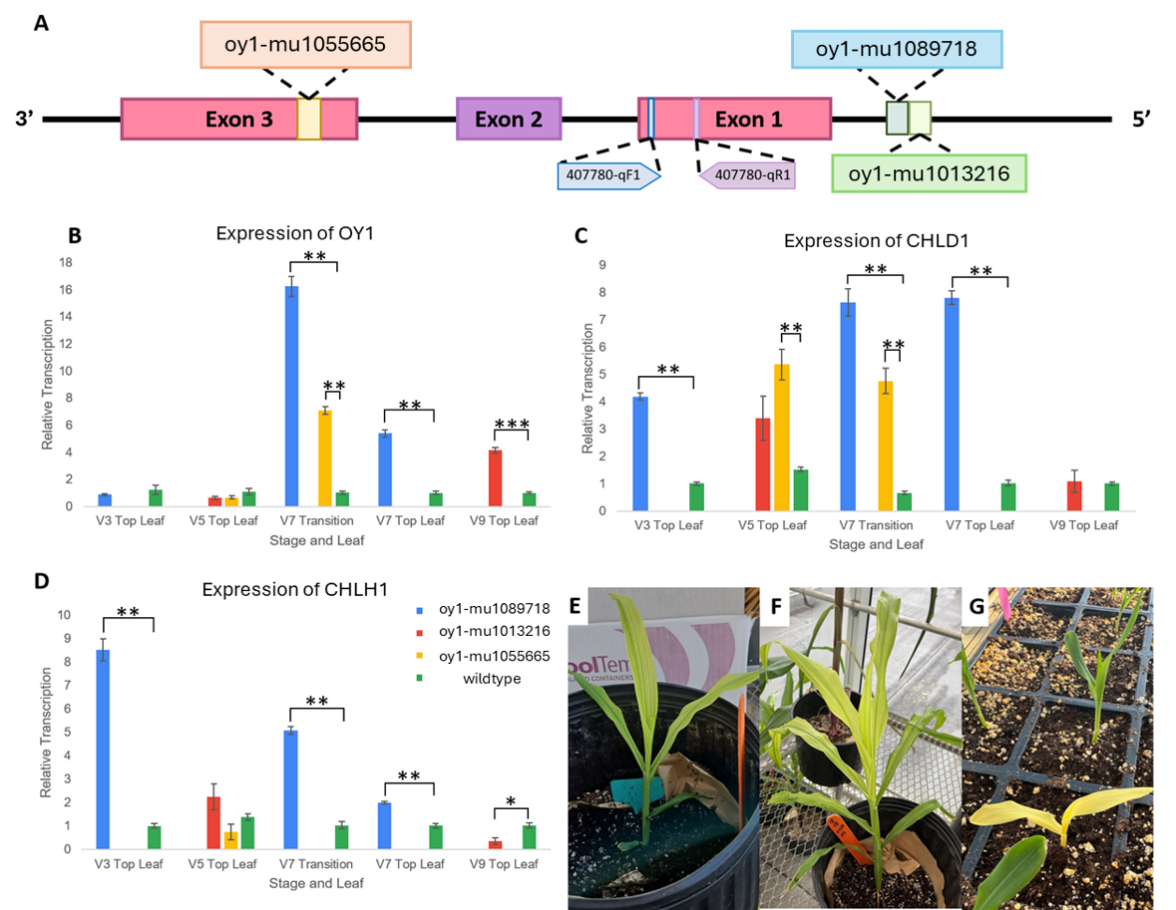
**A:** Schematic representation of the *oy1* gene structure with *Mu* insertions. The *oy1-mu1055665* is located in exon 3, while *oy1-mu1089718* and *oy1-mu1013216* are in the 5′ upstream flanking region, with dashed triangles indicating the location of Targeted Site Duplications (TSDs). Arrows indicate primers used for qRT-PCR of the OY1 transcript **. B–D:** Relative expression levels of OY1 ( **B** ), CHLD1 ( **C** ), and CHLH1 ( **D** ) in wild-type (WT) and *Mu* insertion lines. Samples were collected at two developmental phases: juvenile (V3 and V5, top leaves) and adult (V7, including transition and topmost leaves, and V9 topmost leaf). Expression levels were compared between insertion lines and WT plants from the same leaf type and developmental stage. Asterisks indicate statistical significance ( *p* < 0.05 as *, *p* < 0.01 as **, and *p* < 0.001 as ***) from the non-parametric Mann-Whitney U test on ∆Ct values (Chen et al., 2006). All data are based on two to three biological replicates and three technical replicates. Error bars indicate ± standard errors. Note: Zygosity of *Mutator* insertions was not determined in the samples used for qPCR. Please see the text for further clarification. **E–F:** Representative images of developmental stages studied in this paper: V3 ( **E** ) and V5 ( **F** ). **G:** Image of an "oil yellow" seedling at an early developmental stage from *oy1-mu1013216* .

## Description

Chlorophyll is a key pigment that enables plants to capture light and convert it into energy through photosynthesis. Its production involves a complex biosynthetic pathway (Mascia and Robertson, 1978; Yuan et al., 2020). The first step in this pathway is the insertion of magnesium into protoporphyrin IX, a reaction carried out by magnesium chelatase (MgChl) (Sawers et al., 2006). In *Zea mays* , the *oil yellow1 (oy1)* gene ( *Zm00001eb407780* ) encodes the I subunit of MgChl. When *oy1* is disrupted, plants often display a range of “oil yellow” phenotypes due to reduced chlorophyll.

In this project, we used the Uniform *Mu* transposon tagging resource to ask how *Mu* insertions in or near the *oy1* gene affect its expression. Because transposons are mobile DNA elements that can disrupt coding regions or regulatory sequences, they are often used in functional genomics by providing knock-out or knock-down mutants (Craig et al., 2002). The Uniform *Mu* resource, created by introducing active *Mu* elements into the W22 inbred line, allows for systematic mutagenesis throughout the maize genome (Settles et al., 2007; McCarty et al., 2009).

Based on previous analyses of *Mu* insertions, we expected that insertions would reduce OY1 expression, especially if they were located in exon regions (disrupting coding sequences) or in the 5′ flanking region (possibly disrupting regulatory elements or inducing epigenetic silencing (Bennetzen, 1996; Lisch, 2002). To test this, we looked at three insertion lines: two independent insertions in the 5′ flanking region ( *oy1-mu1013216* : TSD sequences CGGGGGAGG and is 20 bp upstream of ATG; *oy1-mu1089718* : TSD sequences AGGAGCGG and is 14 bp upstream of ATG) and one in exon 3 ( *oy1-mu1055665* : TSD sequences GCCCCACGA and is 478 bp downstream of ATG). The plants tested for each line carried at least one copy of the insertion (see "PCR results for the insertion junctions in all the tested lines" in Extended Data); zygosity (homozygous or heterozygous) was not determined. We measured OY1 transcript levels at two developmental stages: juvenile (V3-V5) and adult (V7-V9).

Our results did not align with our initial hypothesis. OY1 expression remained unchanged during the juvenile stage across all insertion lines, but in the adult phase, expression was significantly upregulated in every tested sample ( **Fig. B** ). Although not all insertions were examined in every tissue or stage, all those tested showed increased expression. In V7 transition leaves, both the flanking insertion *oy1-mu1089718* ( *p* = 0.004998) and the exon insertion *oy1-mu1055665* ( *p* = 0.001756) showed significant upregulation. In the V7 topmost leaf, *oy1-mu1089718* showed increased expression ( *p* = 0.002165); no exon insertion was tested at this stage. At the V9 stage, *oy1-mu1013216* was the only insertion tested and showed significant upregulation in leaf 11 ( *p* = 0.0004).

To determine whether the increase in OY1 expression affected other genes in the same biosynthetic pathway, we examined two additional genes encoding MgChl subunits: *chld1* and *chlh1.* CHLD1 showed a similar pattern in upregulation across most developmental stages ( **Fig. C** ). At the V3 stage, *oy1-mu1089718* ( *p* = 0.004998) exhibited significant upregulation, while no exon insertion was tested. At V5, only the exon insertion *oy1-mu1055665* showed a significant increase in expression ( *p* = 0.004329), with no significant changes observed for the flanking insertions. By V7, the flanking insertion *oy1-mu1089718* was upregulated in both the transition and topmost leaves ( *p* = 0.002165), and the exon insertion *oy1-mu1055665* was significantly increased in the transition leaf ( *p* = 0.001166). At V9, the flanking insertion *oy1-mu1013216* did not exhibit any significant change in expression in leaf 11 ( *p* = 0.328). Although CHLD1 followed a similar trend to OY1, the magnitude of its upregulation was generally smaller.

CHLH1 also responded to the insertions, though the expression pattern was more variable ( **Fig. D** ). At V3, the flanking insertion *oy1-mu1089718* ( *p* = 0.002165) showed strong upregulation; no exon insertion was tested at this stage. At V5, no significant differences in CHLH1 were observed in any of the tested lines. By V7, the flanking insertion *oy1-mu1089718* showed increased expression in both transition and topmost leaves ( *p* = 0.002165), while the exon insertion *oy1-mu1055665* also exhibited significant upregulation in the transition leaf ( *p* = 0.004329). At the V9 stage, *oy1-mu1013216* was significantly downregulated in leaf 11 ( *p* = 0.01199). Overall, CHLH1 showed some early-stage upregulation, but this effect decreased over time.

Although our results did not support the original hypothesis, they were consistent across insertion lines and replicates. Experimental reliability was ensured through at least two biological replicates and three technical replicates per insertion, with W22 serving as the wild-type control throughout. Each insertion line was independently tested by different student groups following a standardized protocol described in “Methods”, with careful monitoring of plant developmental stages.

A significant limitation of this study arises from analyzing plants with undetermined zygosity. While we confirmed the presence of transposon insertions, we did not distinguish between heterozygous and homozygous plants. Consequently, the reported expression levels potentially reflect combined effects from plants of both zygosity states, making these findings preliminary. This limitation directly impacts phenotype interpretation. In Figure 1G, we observed lethal phenotypes, a pattern consistent with homozygous lethal insertions. However, without zygosity testing, we cannot confirm whether these phenotypes correlate with homozygosity. Moreover, plants with exon insertions that were expected to display observable phenotypes instead appeared comparable to wildtype, potentially suggesting heterozygous genotypes. Nevertheless, the absence of zygosity testing prevents a definitive explanation of these observations. Future studies should analyze these groups separately to establish definitive conclusions.

In addition to zygosity testing, future studies should examine alternative splicing patterns. The insertions may disrupt normal splicing, potentially leading to aberrant transcripts that could inflate qRT-PCR measurements or even produce non-functional proteins. Although our analysis of pathway genes (CHLD1 and CHLH1) showed parallel expression patterns that correlated with elevated OY1 transcript levels, direct evidence requires comprehensive transcript characterization through cDNA cloning and sequencing. Protein-level validation would further strengthen these correlations.

This study demonstrates that transposon insertion alleles require thorough transcription analysis rather than assumptions based solely on insertion location. The complex interactions between transposon insertions and gene expression showcase how mobile elements may influence plant gene regulation during development.

## Methods

*Plant Materials and Growth* Maize lines UFMu-07358 ( *oy1-mu1055665* ), UFMu-12181 ( *oy1-mu1089718* ), UFMu-02208 ( *oy1-mu1013216* ), and a wildtype line W22 were obtained from the Maize Genetics Cooperation Stock Center. Seeds were germinated in small pots containing Premier B10281RG ProMix. At approximately the V2 developmental stage, seedlings were transplanted into larger pots to support continued shoot and root development. A diluted 10-10-10 (N-P-K) all-purpose fertilizer was applied at the time of transplanting and subsequently once per week. Leaf color phenotypes potentially associated with altered chlorophyll production were visually monitored twice weekly throughout the growth stages.

*Plant Genotyping*

Genomic DNA was extracted from maize leaf tissue at the V1 developmental stage using the Quick-DNA™ Plant/Seed Miniprep Kit (Zymo Research D6020), following the manufacturer’s protocol.

To identify plants containing *Mutator* ( *Mu* ) transposon insertions in the *oy1* gene, PCR was conducted using PCR Master Mix (Sydlabs, MB067-EQ2B). Each reaction included a primer specific to the *Mu* element (TIR6) and a second primer complementary to the flanking genomic region (see “Reagent” for primer sequences). Tubulin primers were used as internal controls to assess DNA quality, while nuclease-free water was included as a negative control to detect contamination. PCR amplification was performed under the following thermal cycling conditions: initial denaturation at 95 °C for 30 seconds; 35 cycles of 95 °C for 30 seconds, 60 °C for 30 seconds, and 72 °C for 30 seconds; followed by a final extension at 72 °C for 5 minutes. Amplified products were separated by electrophoresis on a 1% agarose gel and visualized using the GelDoc Go Imaging System (Bio-Rad).

*Gene expression assay*

*RNA extraction*

For plants confirmed to carry *Mu* insertions, leaf tissues were collected from two developmental stages: the juvenile stage (prior to V6, e.g., V4 and V5) and the adult stage (V6 or later, e.g., V7 and V9). Two-inches of tissue from the leaf tips were flash-frozen in liquid nitrogen, ground to a fine powder, and stored in TRIzol reagent (Invitrogen) until extraction. Total RNA was extracted using the Direct-zol™ RNA Miniprep Plus Kit (Zymo Research, R2072), following the manufacturer’s instructions including the DNase I treatment.

RNA quality was initially assessed using the Qubit™ RNA IQ Assay (Invitrogen), and concentration was measured by the Qubit™ RNA BR Assay Kit (Thermo Fisher Scientific). RNA integrity and quantity were further verified by electrophoresis on a 1.5% agarose gel. Only samples with high RNA integrity were used in downstream analyses.

*Quantitative Reverse Transcription PCR (qRT-PCR)* Reverse transcription and real-time PCR were performed on high-quality RNA samples in a single step using the Luna® Universal One-Step RT-qPCR Kit (E3005X; New England Biolabs) following the manufacturer’s protocol. Reactions were carried out on the CFX Connect Real-Time PCR Detection System (Bio-Rad).

Transcript levels of three genes, OY1, CHLD1 and CHLH1, were measured, with UBIQUITIN used as internal control. Relative gene expression was calculated using the ΔCt method (Livak and Schmittgen, 2001), with expression levels normalized to UBIQUITIN and compared to W22 wildtype controls at the same developmental stage. For each genotype, three biological replicates and three technical replicates were included. Average relative expression values and standard errors were calculated and reported in the corresponding figure. Statistical significance was determined using the Wilcoxon two-sample test on ΔCt values, as described by Yuan et al. (2006).

## Reagents

**Table:** 

Purpose	Primer Name	5’ to 3’ sequences	Regions
Genotyping	SLSW_R2	CCCCCGCTCCTTCCACACAAAC	Flanking primer for *oy1-mu1055665*
	GAVF_F2	CGTTGAGCAGCAGGCAGAGCTT	Flanking primer for *oy1-mu1089718* / *1013216*
	TIR6	AGAGAAGCCAACGCCAWCGCCTCYATTTCGTC	*Mu* sequences
	Tub_F	CTACCTCACGGCATCTGCTATGT	Internal control
	Tub_R	GTCACACACACTCGACTTCACG	Internal control
qRT-PCR	ubi-tFor	GTCATAGTTCTGGGTAGTACGC	Internal control
	ubi-tRev	TGGAGGTTGTCAAAGTATCTGC	Internal control
	407780-qF1	CTGAAGCGGCGAGGAAGAG	OY1
	407780-qR1	AGAAGGTGGAAGCCATGACG	OY1
	chld1_qF	TGCTGAGGTTTTGCTTCCAC	CHLD1
	chld1_qR	ACATCGCCACTCTTTTCAGC	CHLD1
	chlh1_qF	CCTCGTACATAGCCGACACC	CHLH1
	chlh1_qR	CGCTTCTCGATCTCCCTGAC	CHLH1

## Data Availability

Description: RealTime PCR results for gene expressions. Resource Type: Dataset. DOI: https://doi.org/10.22002/6d6hx-4ac19 Description: Genotyping Results. Resource Type: Dataset. DOI: https://doi.org/10.22002/n2bpt-8wv27
